# Investigation of the regulatory mechanisms of Guiqi Yimu Powder on dairy cow fatty liver cells using a multi-omics approach

**DOI:** 10.3389/fvets.2024.1475564

**Published:** 2024-10-09

**Authors:** Chenlei Li, Feifei Wang, Yanfen Ma, Wenjia Wang, Yansheng Guo

**Affiliations:** ^1^College of Animal Science and Technology, Ningxia University, Yinchuan, China; ^2^Key Laboratory of Ruminant Molecular and Cellular Breeding of Ningxia Hui Autonomous Region, College of Animal Science and Technology, Ningxia University, Yinchuan, China

**Keywords:** Guiqi Yimu Powder, dairy cow, fatty liver, multi-omics, network pharmacology

## Abstract

**Introduction:**

Fatty liver disease in dairy cows is a metabolic disorder that significantly affects their health and productivity, imposing a notable economic burden on the global dairy industry. Traditional Chinese medicine (TCM), characterized by its multi-component and multi-target features, has shown unique advantages in the prevention and treatment of various diseases. Guiqi Yimu Powder, a traditional TCM formula, enhances growth, boosts production efficiency, and strengthens immune function in livestock by regulating antioxidant along with anti-inflammatory pathways. However, its specific regulatory mechanisms on fatty liver in dairy cows remain unclear. This study aims to investigate the molecular-level effects and potential regulatory mechanisms of Guiqi Yimu Powder in a Trimethylamine N-oxide (TMAO) induced fatty liver cell model of dairy cows.

**Methods:**

We employed a comprehensive analysis integrating transcriptomics, proteomics, metabolomics, and network pharmacology. An *in vitro* dairy cow fatty liver cell model was established using TMAO to induce lipid accumulation. Cells were treated with the optimal TMAO concentration identified through preliminary experiments, and further divided into a lipid accumulation group and Guiqi Yimu Powder treatment groups. The treatment groups received varying concentrations of Guiqi Yimu Powder (10, 20, 30, 40, or 50 g/L). High-throughput omics sequencing technologies were utilized to perform a comprehensive analysis of the treated cells. Bioinformatics methods were applied to explore the regulatory effects, aiming to elucidate the specific impacts of Guiqi Yimu Powder on lipid metabolism, liver function, and related signaling pathways, thereby providing scientific evidence for its potential application in the prevention and treatment of fatty liver in dairy cows.

**Results:**

Guiqi Yimu Powder treatment significantly affected 1,536 genes, 152 proteins, and 259 metabolites. KEGG enrichment analysis revealed that the significantly altered molecules are involved in multiple pathways related to the pathology of fatty liver, including metabolic pathways, glutathione metabolism, hepatitis B, and AMPK signaling pathway (*p* < 0.05). Notably, joint analysis highlighted the regulatory mechanisms of Guiqi Yimu Powder on glutathione cycling, with L-5-Oxoproline identified as an important metabolic compound. These findings indicate its impact on oxidative stress, energy metabolism, and liver function, suggesting potential therapeutic applications for fatty liver in dairy cows.

**Discussion:**

This study elucidated the regulatory mechanisms of Guiqi Yimu Powder on fatty liver cells in dairy cows, providing new scientific evidence for its potential application in the prevention and treatment of fatty liver disease.

## 1 Introduction

In the dairy farming industry, fatty liver is a severe metabolic disorder that frequently occurs, especially among groups of high-yield dairy cows. The periparturient period (from 3 weeks before to 3 weeks after calving) is a high-risk time for the development of fatty liver due to the increased metabolic load, which disrupts lipid metabolism and leads to abnormal accumulation of fat in the liver (1–3). It is estimated that the incidence of fatty liver can reach up to 30% in high-yield dairy cows (4). Fatty liver not only deteriorates the overall health of dairy cows, making them more susceptible to diseases such as mastitis, hoof diseases, and reproductive problems, but also directly affects milk production and quality. This not only brings direct economic losses to the dairy farming industry but also poses significant challenges in reducing production costs and enhancing market competitiveness (5). Studies report that metabolic diseases related to fatty liver, such as subclinical ketosis (SCK), can result in economic losses of approximately €130 per case per year. These losses stem from decreased milk production, increased treatment costs, extended calving intervals, and higher culling rates (6). Therefore, exploring effective prevention and treatment methods is crucial for the dairy farming industry. TMAO is a common nutrient, primarily produced from dietary choline, phosphatidylcholine, and L-carnitine through the action of gut microbiota, which generate trimethylamine (TMA). Subsequently, TMA is oxidized to TMAO in the liver by flavin monooxygenases (FMO1 and FMO3) (7). TMAO influences the development of fatty liver disease through multiple mechanisms. It has been reported that TMAO regulates lipid metabolism by affecting cholesterol metabolism, lipoprotein metabolism, and fatty acid oxidation (8–12). Additionally, TMAO upregulates the expression of inflammation-related genes, promotes the release of inflammatory cytokines, and induces a sustained inflammatory response in the liver. Moreover, TMAO increases the production of reactive oxygen species (ROS), leading to oxidative stress, which further disrupts the integrity of hepatocyte membranes and induces apoptosis (13). Given the crucial role of TMAO in regulating lipid metabolism, inflammatory responses, and oxidative stress, constructing a dairy cow fatty liver cell model centered on TMAO can effectively reveal the pathological mechanisms and provide theoretical and experimental foundations for the development of targeted prevention and treatment strategies (14).

Guiqi Yimu Powder is a TCM formulation widely used in veterinary medicine, composed mainly of Angelica sinensis (Danggui), Astragalus membranaceus (Huangqi), and Leonurus japonicus (Yimucao). Its primary functions are to promote blood circulation, remove blood stasis, and tonify Qi and blood, making it widely used for treating conditions such as retained placenta and endometritis in postpartum livestock, which are attributed to Qi deficiency and blood stasis (15). Each component of the formula plays a crucial role: Danggui is known for its abilities to nourish and invigorate blood, regulate menstruation, and alleviate pain (16); Huangqi excels in tonifying Qi, raising Yang, and stabilizing the exterior to stop sweating (17); Yimucao is renowned for promoting blood circulation, removing stasis, and reducing swelling through diuresis (18). The synergistic effects of these herbs make Guiqi Yimu Powder highly effective in treating disorders related to blood stasis and Qi deficiency. In recent years, numerous studies have reported that Guiqi Yimu Powder can enhance growth performance, production performance, and immune function in livestock and poultry through various mechanisms, including antioxidation, anti-inflammation, and immune regulation (19). Additionally, it has been shown to modulate the rumen environment of dairy cows and alleviate liver damage (20, 21). As research continues to advance, the application prospects of Guiqi Yimu Powder are becoming increasingly broad and diverse, and its value in the livestock industry is being progressively recognized and appreciated.

In recent years, with the rapid advancement of high-throughput sequencing technologies, omics research methods such as transcriptomics, proteomics, and metabolomics have been widely applied in biomedical and agricultural sciences. These methods provide powerful tools for uncovering various physiological regulatory mechanisms. For instance, Mao Yongxia et al. utilized UPLC-MS/MS lipidomics to discover that Guiqi Yimu Powder might exert its anti-inflammatory and antioxidant effects by regulating metabolic pathways such as arachidonic acid metabolism, TRP channel-mediated inflammatory mediators, and the PPAR signaling pathway in postpartum dairy cows (20). Turner et al., using proteomics, found that negative energy balance (NEB) is associated with significant changes in the liver proteome of early lactating dairy cows, including increased fatty acid uptake, impaired anti-inflammatory responses, and mitochondrial dysfunction, providing potential disease biomarkers and therapeutic targets (22). Gao et al. conducted transcriptomic analysis on the livers of high-yield dairy cows during the transition from pregnancy to lactation, revealing the activation of metabolic pathways, particularly significant changes in lipid, glucose, and amino acid metabolism, as well as the crucial roles of PPAR and adipocytokine signaling pathways in this adaptive process (23). Nonetheless, studies based on a single perspective often fall short in fully explaining complex biological phenomena. Moreover, the application of these technologies in TCM research within the livestock industry is still limited. For complex TCM formulations, a systematic multi-omics analysis can provide a more comprehensive and in-depth understanding.

This study integrates transcriptomic, proteomic, and metabolomic data to investigate the regulatory mechanisms of Guiqi Yimu Powder on dairy cow fatty liver cells from a systems biology perspective. By analyzing the complex interactions between Guiqi Yimu Powder and the regulatory mechanisms of fatty liver cells, we aim to provide new theoretical foundations for the prevention and treatment of fatty liver in dairy cows.

## 2 Materials and methods

### 2.1 Preparation and identification of Guiqi Yimu Powder

Guiqi Yimu Powder (Beijing Edison Biotechnology Co., Ltd., batch number: 2103264) was composed of Angelica sinensis, Astragalus membranaceus, and *Leonurus japonicus* in a ratio of 2:2:1. Each 1 mL of the extract corresponds to 0.5 g of the raw medicinal herb. During preparation, raw materials underwent selection and quality control to ensure their active ingredients and purity met the required standards. The herbs were then washed, dried, and coarsely ground for solvent extraction. The extract was filtered and concentrated under reduced pressure using a rotary evaporator to remove most of the solvent. Subsequently, the concentrated extract was converted into powder using vacuum freeze-drying technology and was standardized to ensure consistency in active ingredients.

The prepared Guiqi Yimu Powder underwent three rounds of ultra-high performance liquid chromatography–tandem mass spectrometry (UHPLC–MS/MS) analysis. For the sample preparation, the medicinal solution was thawed at 4°C and vortex mixed until uniform. A 1 mL aliquot of the solution was mixed with 3 mL of absolute ethanol, vortexed for 3 min, and subjected to ultrasonication in an ice bath for 10 min. The mixture was left to stand at room temperature for 12 h, followed by centrifugation at 7,000 g for 20 min at 4°C. The supernatant was collected and dried under nitrogen to obtain the refined extract. For mass spectrometry, the dried extract was reconstituted in ultrapure water to a final volume of 1 mL and filtered through a 0.22 μm membrane. Throughout the analysis, samples were kept in a 4°C autosampler. Chromatographic separation was performed using a SHIMADZU LC-30 UHPLC system equipped with an ACQUITY UPLC^®^ HSS T3 column (2.1 × 100 mm, 1.8 μm) from Waters (Milford, MA, United States). Electrospray ionization (ESI) was employed for both positive (+) and negative (−) ion modes. Post-UPLC separation, mass spectrometric analysis was conducted on a QE Plus mass spectrometer (Thermo Scientific) utilizing a heated electrospray ionization (HESI) source. The total run time for each sample was 15 min. The mass range for the parent ion scan was set to 75–1,050 m/z. Raw data were processed using MSDIAL software for peak alignment, retention time correction, and peak area extraction. Metabolite structures were identified by matching accurate mass values (mass tolerance <0.01 Da) against a TCM database.

### 2.2 Construction and functional analysis of the active compound-target network of Guiqi Yimu Powder

In this study, we utilized the Traditional Chinese Medicine Systems Pharmacology (TCMSP) database^1^ to identify all the compounds in Guiqi Yimu Powder, which includes Astragalus membranaceus, *Leonurus japonicus*, and Angelica sinensis (24). Literature searches and database queries were performed based on the chemical components identified from the UHPLC–MS/MS analysis. A compound-target network for Guiqi Yimu Powder was constructed using Cytoscape v3.10.1 software, incorporating the screened compounds and their corresponding target information. To construct a protein–protein interaction (PPI) network, the target genes associated with Guiqi Yimu Powder were uploaded to the STRING database (v12.0)^2^ to investigate the interactions between target proteins (25). Furthermore, we used ClueGo software to perform a Kyoto Encyclopedia of Genes and Genomes (KEGG) pathway enrichment analysis on the proteins and genes within the PPI network. These analyses are intended to elucidate the mechanisms by which Guiqi Yimu Powder treats bovine fatty liver at the levels of gene function and signaling pathways (26, 27).

### 2.3 Dairy cow hepatocyte culture and treatment

Dairy cow hepatocytes were provided by Professor Yanfeng Ma from Ningxia University and were purchased from BLUEFBIO (Cat# BFN60810761, Shanghai, China). The cell density was adjusted to 1 × 10^6^ cells/mL and seeded into T75 culture flasks. The culture medium consisted of Dulbecco’s Modified Eagle Medium (DMEM, Cat# c11995500BT, Thermo Fisher Scientific, Beijing, China), 10% fetal bovine serum (FBS, Cat# 1099-141, Thermo Fisher Scientific, Australia), and 1% penicillin–streptomycin. The cells were incubated at 37°C in a humidified atmosphere containing 5% CO_2_. When the cells reached approximately 80% confluence, the medium was replaced with serum-free DMEM for a further 12 h of incubation.

For treatment, the medium was replaced, and cells were divided into groups. The control group received fresh complete medium, while experimental groups were exposed to DMEM with 200, 300, or 400 μmol/L TMAO (Cat# T833724-1 g, Macklin Biochemical Co, Ltd., Shanghai, China), for 12 h to establish the optimal lipid-inducing concentration of TMAO, determined via liver function assays. Subsequently, hepatocytes at stable passage underwent the aforementioned starvation protocol and were randomly assigned into new groups. The lipid accumulation group (Group A) received DMEM with the optimal TMAO concentration. Guiqi Yimu Powder treatment groups (Groups B1–B5) were supplemented with the same TMAO concentration and varying concentrations of Guiqi Yimu Powder (10, 20, 30, 40, 50 g/L). Cells were incubated for an additional 12 h under identical conditions. Post-treatment, cell pellets were harvested, flash-frozen in liquid nitrogen for 30 min, and stored at −80°C for further analysis.

### 2.4 Biochemical detection of liver injury and lipid metabolism markers, and Oil Red O staining

The collected cell pellets were lysed using an ultrasonic homogenizer. The levels of aspartate aminotransferase (AST), alanine aminotransferase (ALT), and triglycerides (TG) in the samples were measured using biochemical assay kits (microplate method, AST: C010-2-1, ALT: C009-2-1, and TG: A110-1-1, Nanjing Jiancheng Bioengineering Institute, Nanjing, China) according to the manufacturer’s instructions. Subsequently, lipid droplets were stained using an Oil Red O Staining Kit (C0158S, Shanghai Beyotime Biotechnology, Shanghai, China). The collected samples were fixed in 10% neutral formalin, followed by dehydration with a series of ethanol solutions. After rehydration, the samples were stained with freshly prepared Oil Red O solution for 15 min. Excess stain was removed by washing with 60% isopropanol, and counterstaining was performed with hematoxylin. The stained samples were then mounted with glycerin gelatin and observed under a microscope to quantify the lipid droplets.

### 2.5 Transcriptomic sequencing and analysis

To ensure the quality of experimental data, we selected three groups of samples for RNA sequencing, with each group consisting of three replicates to enhance the reliability of the results and ensure statistical significance. The RNA-seq services were provided by Shanghai Bioprofile Co, Ltd. (Shanghai, China). Total RNA was extracted from the collected cell samples using Trizol reagent (Cat# CW0580, CWbio, Jiangsu, China). The concentration, quality, and integrity of the RNA were then determined using a NanoDrop spectrophotometer (Thermo Scientific). The constructed libraries were purified using the AMPure XP system (Beckman Coulter, Beverly, CA, United States) and quantified using the Agilent High Sensitivity DNA Kit on the Agilent 2100 Bioanalyzer system. Finally, high-throughput sequencing of the libraries was conducted on the Illumina NovaSeq 6000 platform.

The raw data in FASTQ format were filtered using Cutadapt (v1.15) software, and the filtered reads were aligned to the reference genome using HISAT2 (v2.0.5) (28). For the quantitative analysis of gene expression levels, HTSeq (v0.9.1) was used to count the read numbers mapped to each gene, which served as the initial expression value (29). These expression values were then normalized using the FPKM (Fragments Per Kilobase of transcript per Million mapped reads) method. Principal Component Analysis (PCA) was performed using the Omicshare data processing platform developed by GENE DENovo^3^ under default settings. Differentially expressed genes (DEGs) were identified using the DESeq (v1.30.0) R package with the criteria |FC| > 1.5 and *p* < 0.05 (30). Clustering analysis of gene expression patterns among samples was conducted using the Pheatmap package (v1.0.8) in R, and the results were visualized as heatmaps (31). Finally, the DEGs were annotated using the KEGG database^4^ (32), and their enrichment was analyzed with the ClusterProfiler package (v3.4.4) (33).

### 2.6 Proteomics sequencing and analysis

To ensure consistency between the proteomics and transcriptomics datasets, we conducted proteomics analysis on the same set of three samples. The proteomics analysis services were provided by Shanghai Bioprofile Co., Ltd. (Shanghai, China). Proteins were extracted from the cell samples (*n* = 3) using SDT lysis buffer (4% SDS, 100 mM DTT, 100 mM Tris–HCl, pH 8.0). The samples were boiled for 5 min and then sonicated, followed by another boiling step for 5 min. After centrifugation (16,000 g, 15 min), the supernatants were collected, and the protein concentrations were determined using the BCA Protein Assay Kit (Bio-Rad, United States). For each sample, 200 μg of protein was digested using the FASP method as described by Wisniewski et al. The resulting peptides were desalted using C18 StageTips prior to LC–MS analysis (34). The peptide concentrations were measured at OD280 using a Nanodrop One device.

LC–MS/MS analysis was performed using a Q Exactive Plus mass spectrometer coupled with an Easy 1,200 nLC system (Thermo Fisher Scientific). Mass spectrometry data were analyzed using MaxQuant software (v1.6.0.16) (35), with protein identification conducted against the UniProtKB *Rattus norvegicus* database. Protein quantification in MaxQuant was carried out using the ratio median normalization method (36). Principal Component Analysis (PCA) of protein expression levels was performed using the Omicshare data processing platform.^5^ Fold changes (FC) in protein abundance and *p*-values were calculated based on LFQ values, with selection criteria set at |FC| > 1.5 and *p* < 0.05. The differentially expressed proteins were annotated and subjected to significant enrichment analysis using the KEGG database (37).

### 2.7 Untargeted metabolomics sequencing and analysis

A larger sample size (*n* = 6) was utilized for metabolomics analysis to capture a broader spectrum of metabolites and ensure sufficient statistical power. These cell samples included the same three samples initially used for transcriptomics and proteomics, along with an additional three samples to enhance the robustness of metabolite detection. The untargeted metabolomics analysis services were provided by Shanghai Bioprofile Co., Ltd. (Shanghai, China). The collected cell samples (*n* = 6) were mixed with 1 mL of pre-cooled methanol/acetonitrile/water (v/v/v, 2:2:1) and sonicated in an ice-water bath for 1 h. The samples were then incubated at −20°C for 1 h to precipitate proteins. After centrifugation at 14,000 g for 20 min (4°C), 800 μL of the supernatant was transferred to sampling tubes. The vacuum-dried extract was reconstituted in 50% acetonitrile, filtered through a 0.22 μm cellulose acetate membrane, and transferred to 2 mL HPLC vials, then stored at −80°C until analysis.

Metabolomics analysis was performed using a UPLC-ESI-Q-TOF-MS system (UHPLC, Shimadzu Nexera X2 LC-30 AD, Shimadzu, Japan) in combination with a Q-Exactive Plus mass spectrometer (Thermo Scientific, San Jose, United States). Liquid chromatography (LC) separation was conducted with an ACQUITY UPLC^®^ HSS T3 column (2.1 × 100 mm, 1.8 μm) (Waters, Milford, MA, United States). The flow rate was set to 0.3 mL/min with mobile phases A (0.1% formic acid in water) and B (100% acetonitrile, ACN). The gradient conditions were: 0% B for 2 min, a linear increase to 48% B over 4 min, an increase to 100% B over the next 4 min, held for 2 min, followed by a rapid decrease to 0% B in 0.1 min, with re-equilibration for 3 min. Electrospray ionization (ESI) was performed in both positive and negative ion modes for mass spectrometry data acquisition. Multivariate data analysis and modeling were performed using R (v4.0.3) and relevant R packages. Principal component analysis (PCA) and orthogonal partial least squares discriminant analysis (OPLS-DA) were used to construct the models (38). OPLS-DA was employed to determine the importance of variables to the projection (VIP), with significant differential metabolites being selected based on VIP ≥ 1 and FC > 1.5 or FC < 0.667, and *p* < 0.05. Clustering analysis of the selected differential metabolites was conducted using R, and KEGG pathway enrichment analysis was performed using the KEGG database to identify key metabolic pathways. For a systematic study of metabolic changes, overall trends in metabolic pathways were analyzed based on the abundance of differential metabolites. The annotation results of the differential metabolites were used to calculate the DA score, and the top 30 pathways were visualized for further insight.

### 2.8 Integrated analysis of transcriptomics, proteomics, and metabolomics

Based on the KEGG pathway enrichment analysis results, which were significantly associated with the regulatory mechanisms of Guiqi Yimu Powder on fatty liver cells (*p* < 0.05), key differential genes, proteins, and metabolites were selected. A multi-omics functional network diagram was created using Cytoscape software (39). Correlation heatmaps were constructed using the Pearson algorithm in R (v4.0.3), and correlation networks were also visualized with Cytoscape software. Finally, we integrated the multi-omics data to construct a conceptual model illustrating the potential impact of TMAO on lipid metabolism in bovine hepatic cells.

### 2.9 Statistical analysis

All data were initially processed using Excel software and subsequently analyzed with GraphPad Prism software (v. 8.0). Analysis of Variance (ANOVA) was performed for the statistical analysis of data results. Pairwise significant differences between groups were evaluated using Duncan’s test. Differences were considered statistically significant when *p* < 0.05 and highly statistically significant when *p* < 0.01.

## 3 Results

### 3.1 Component analysis of Guiqi Yimu Powder

**Figure 1:**
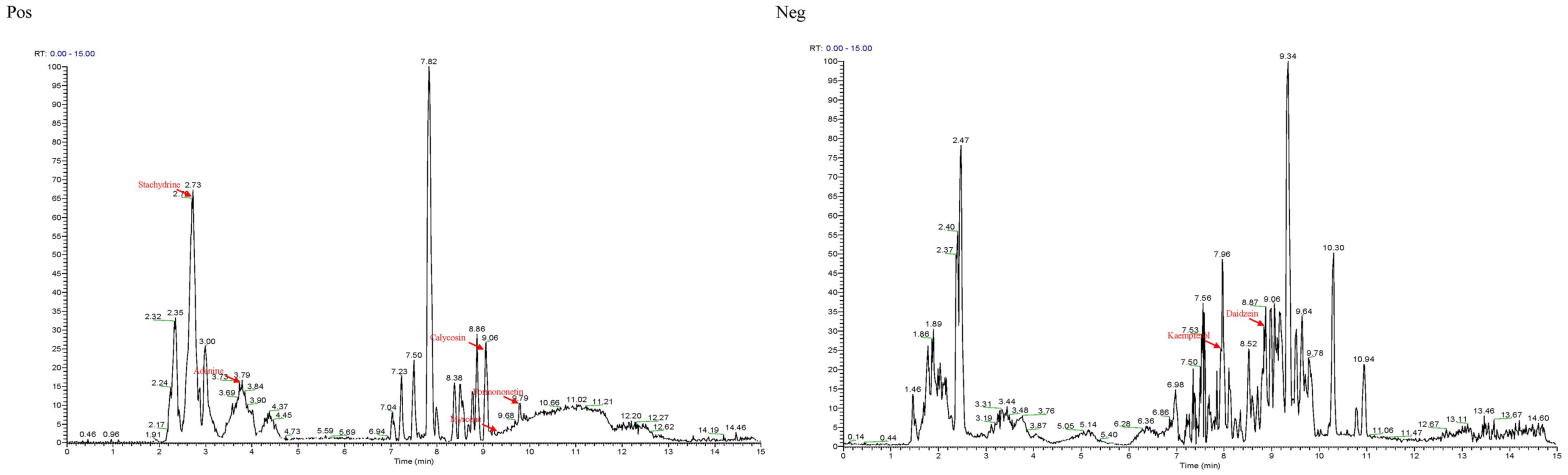
The base peak ion chromatogram of Guiqi Yimu Powder samples by UHPLC–MS/MS analysis.

The total ion chromatograms of Guiqi Yimu Powder were shown in Figure 1. By comparing the UHPLC–MS/MS results with the precursor ion mass-to-charge ratios, secondary spectra, or retention times of standard substances or database entries, a total of 348 chemical components were identified in Guiqi Yimu Powder (Supplementary Table S1). These components mainly include flavonoids, phenols, carboxylic acids and their derivatives, steroids and their derivatives, fatty acids and their derivatives, hydroxy acids and their derivatives, indoles and their derivatives, among others.

Notably, among the 348 compounds, 7 major components were identified: Daidzein, Kaempferol, Adenine, Myrcene, Calycosin, Formononetin, and Stachydrine. These 7 compounds are the primary constituents of Angelica sinensis, Astragalus membranaceus, and *Leonurus japonicus*, as marked in Figure 1. Consequently, it can be inferred that these compounds reflect the major extract components of the three medicinal plants present in Guiqi Yimu Powder.

### 3.2 Construction and functional analysis of the target PPI network for Guiqi Yimu Powder

**Figure 2:**
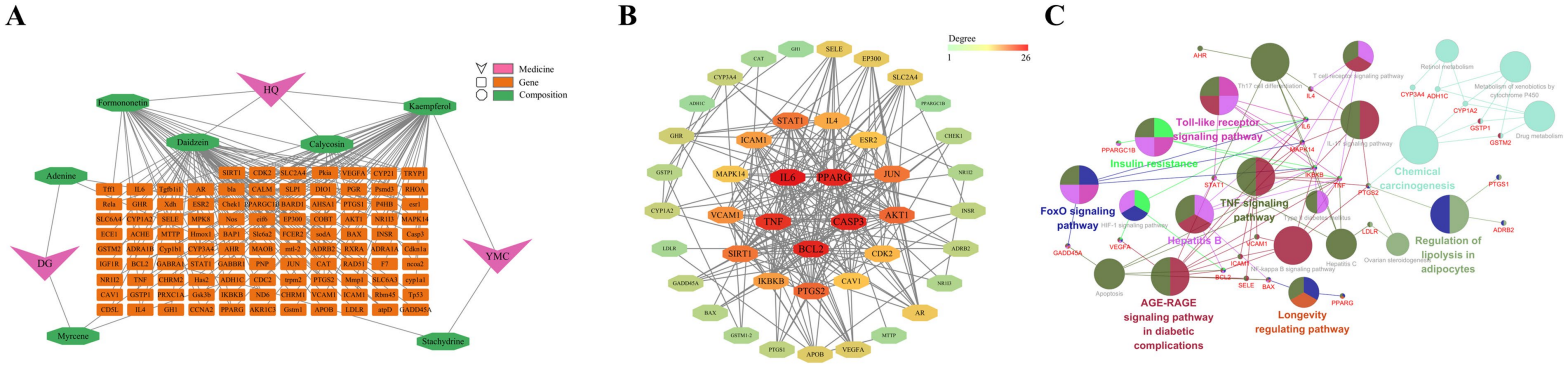
Construction and functional analysis of the target PPI network for Guiqi Yimu Powder. **(A)** Composite target network of intersecting genes from Guiqi Yimu Powder’s active components and disease target databases. **(B)** PPI network of intersecting genes. **(C)** Network diagram of KEGG enrichment analysis for intersecting genes.

All the chemical components present in Astragalus membranaceus, *Leonurus japonicus*, and Angelica sinensis within Guiqi Yimu Powder were retrieved using the TCMSP database. We screened and identified seven active components: four from Astragalus membranaceus, two from Angelica sinensis, and two from *Leonurus japonicus*, where kaempferol is a shared component of both Astragalus membranaceus and *Leonurus japonicus*. These active components correspond to 106 potential target genes. A regulatory network illustrating these interactions was then constructed (Figure 2A), providing a comprehensive visualization of the interactions between Guiqi Yimu Powder’s active components and their corresponding targets.

Gene data related to the pathogenesis of fatty liver disease were collected from the GeneCards and DisGeNet databases. After removing redundant targets, 11,571 disease-related targets were identified. By intersecting the 106 target genes of Guiqi Yimu Powder’s active components with these disease-related genes, we identified 43 potential therapeutic targets for the powder. To further understand the mechanism of action of Guiqi Yimu Powder, we used STRING software to construct a PPI network for these intersected genes (Figure 2B). The PPI network revealed extensive interactions among these genes’ proteins. By analyzing the network’s topology, we identified several core proteins occupying key positions within the network, such as *IL6*, *TNF*, *PPARG*, *CASP3*, and *BCL2*. These proteins were likely to play crucial roles in Guiqi Yimu Powder’s treatment of fatty liver. Figure 2C showed the major signaling pathways identified through KEGG enrichment analysis (*p* < 0.05). The genes were found to be involved in multiple pathways, including lipid metabolism pathways like the Regulation of lipolysis in adipocytes and the Insulin resistance, inflammation-related pathways like the IL-17 signaling pathway and the NF-κB signaling pathway, and cell proliferation and apoptosis pathways such as the Apoptosis pathway and Longevity regulating pathway. These pathways are critical in the development and progression of fatty liver. Through the construction and analysis of the target PPI network for Guiqi Yimu Powder, we uncovered its multi-target and multi-pathway synergistic mechanisms in treating fatty liver. These findings provide a theoretical basis for its effective clinical application.

### 3.3 Analysis of TMAO-induced hepatocyte injury and lipid droplet formation in bovine cells

**Figure 3:**
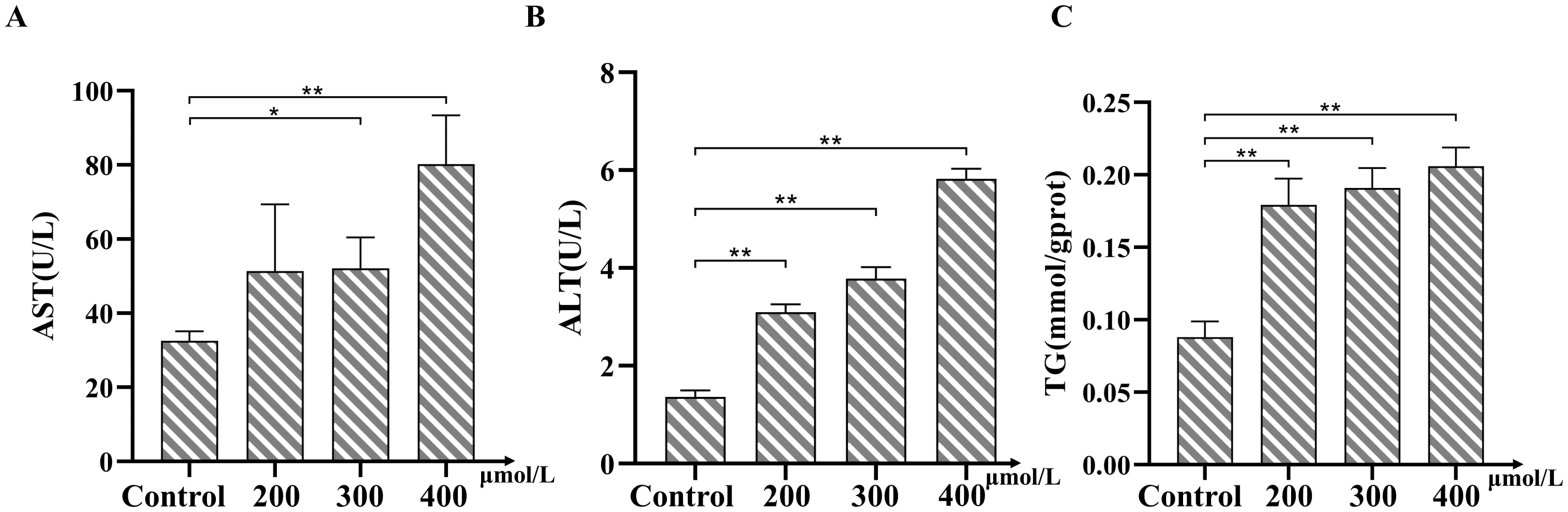
Effects of Trimethylamine-N-oxide (TMAO) on AST, ALT, and TG levels in bovine hepatocytes. **(A)** Changes in aspartate aminotransferase (AST) levels under different concentrations of TMAO treatment. **(B)** Changes in alanine aminotransferase (ALT) levels under different concentrations of TMAO treatment. **(C)** Changes in TG levels under different concentrations of TMAO treatment. Compared to the control group, * indicates *p* < 0.05, ** indicates *p* < 0.01.

In this study, we investigated the effects of various concentrations of TMAO on bovine hepatocytes. We assessed the extent of hepatocyte injury by measuring the intracellular levels of AST, ALT, and TG. The experimental results indicated that TMAO elevated the levels of AST, ALT, and TG at all tested concentrations. Notably, AST levels significantly increased at concentrations of 300 μmol/L and 400 μmol/L (*p* < 0.05, Figure 3A). Similarly, ALT and TG levels showed significant increases following treatment with different TMAO concentrations (*p* < 0.05, Figures 3B,C). These data suggest that TMAO induces hepatocyte injury across all tested concentrations, with the most pronounced increases in cell injury and TG levels observed at a concentration of 400 μmol/L.

### 3.4 Effects of Guiqi Yimu Powder on lipid accumulation in bovine hepatocytes

**Figure 4:**
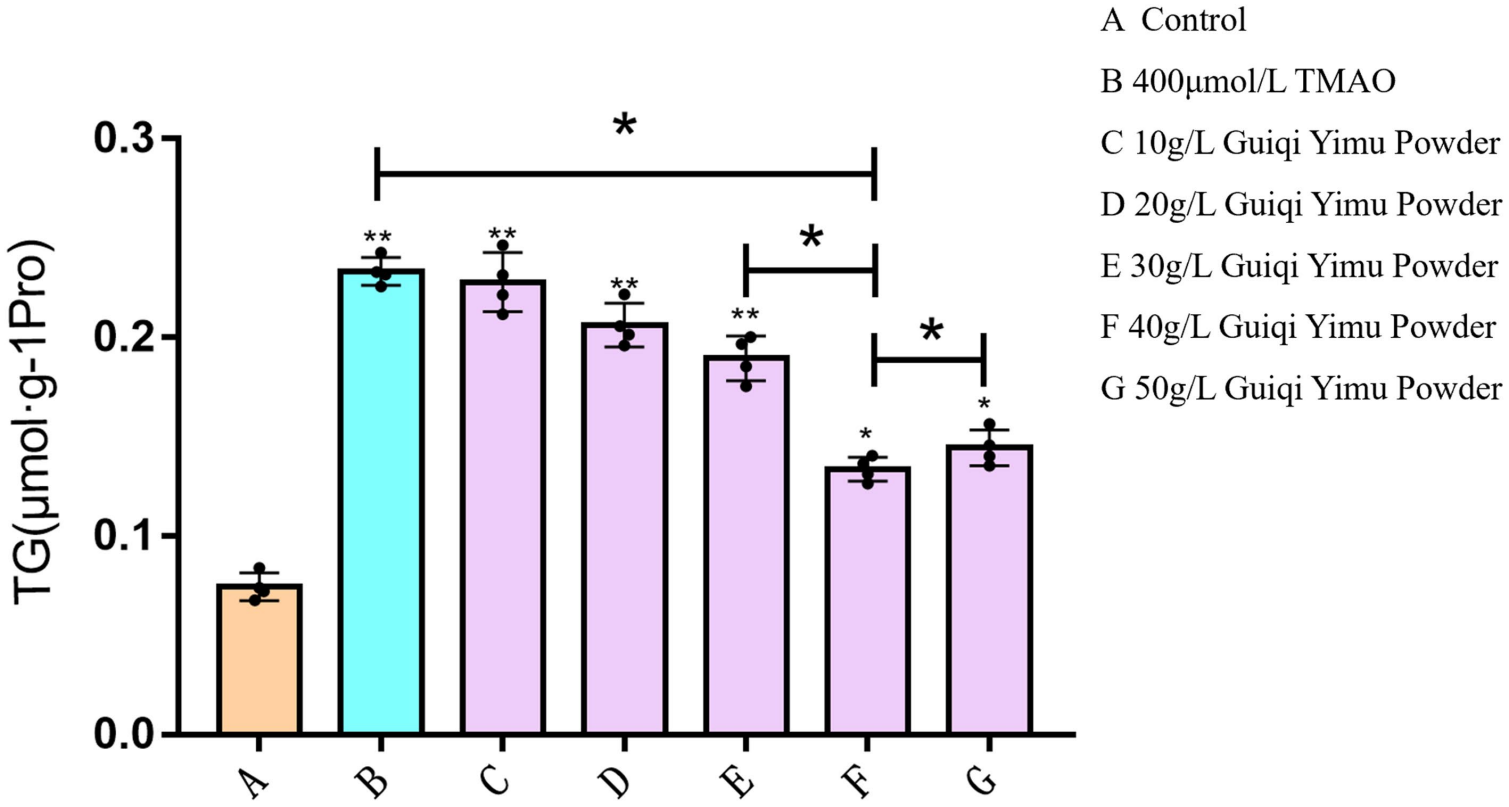
Effects of Guiqi Yimu Powder on TG levels in hepatocytes with lipid accumulation.

Next, we investigated the ameliorative effects of Guiqi Yimu Powder on TMAO-induced hepatocyte injury and lipid droplet accumulation. As shown in Figure 4, TG levels were significantly elevated (*p* < 0.01) in the group with lipid accumulation induced by TMAO at the optimal concentration compared to the control group. Treatment with Guiqi Yimu Powder at concentrations of 10 g/L, 20 g/L, and 30 g/L significantly reduced TG levels in hepatocytes with fatty liver (*p* < 0.01). The lowest TG levels were observed in the group treated with 40 g/L Guiqi Yimu Powder, which also showed a significant difference compared to the previous group. Thus, a concentration of 40 g/L of Guiqi Yimu Powder was most effective in reducing TG levels and could be considered the optimal concentration for preventing hepatocyte lipid accumulation.

**Figure 5:**
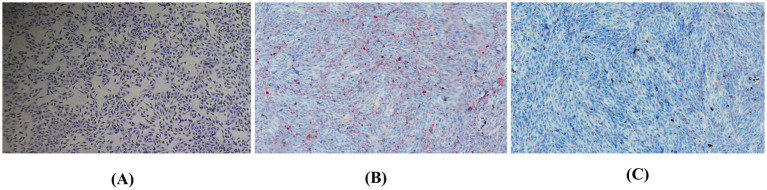
Oil Red O staining showing the effects of Guiqi Yimu Powder treatment on lipid accumulation in bovine hepatocytes. **(A)** Oil Red O staining of the control group bovine hepatocytes. **(B)** Oil Red O staining of bovine hepatocytes treated with 400 μmol/L TMAO. **(C)** Oil Red O staining of bovine hepatocytes treated with 40 g/L Guiqi Yimu Powder.Oil Red O staining was used to visualize the accumulation of intracellular lipid droplets, with red regions representing lipid droplets.

To further elucidate the impact of TMAO on lipid accumulation in hepatocytes, we performed Oil Red O staining with 400 μmol/L TMAO. In the control group, bovine hepatocytes displayed deep blue nuclei with sparse red lipid droplets scattered in the cytoplasm (Figure 5A). However, treatment with 400 μmol/L TMAO resulted in a substantial accumulation of dispersed red lipid droplets in the cytoplasm (Figure 5B). Compared to the TMAO-only group, the Guiqi Yimu Powder treatment group showed a significant reduction in red lipid droplets within the cytoplasm (Figure 5C). In summary, a concentration of 400 μmol/L TMAO caused significant hepatocyte injury and lipid droplet accumulation in bovine hepatocytes In contrast, Guiqi Yimu Powder markedly mitigates TMAO-induced hepatocyte injury and lipid droplet accumulation, demonstrating its potential protective effects.

### 3.5 Identification and comparison of differentially expressed genes

**Figure 6:**
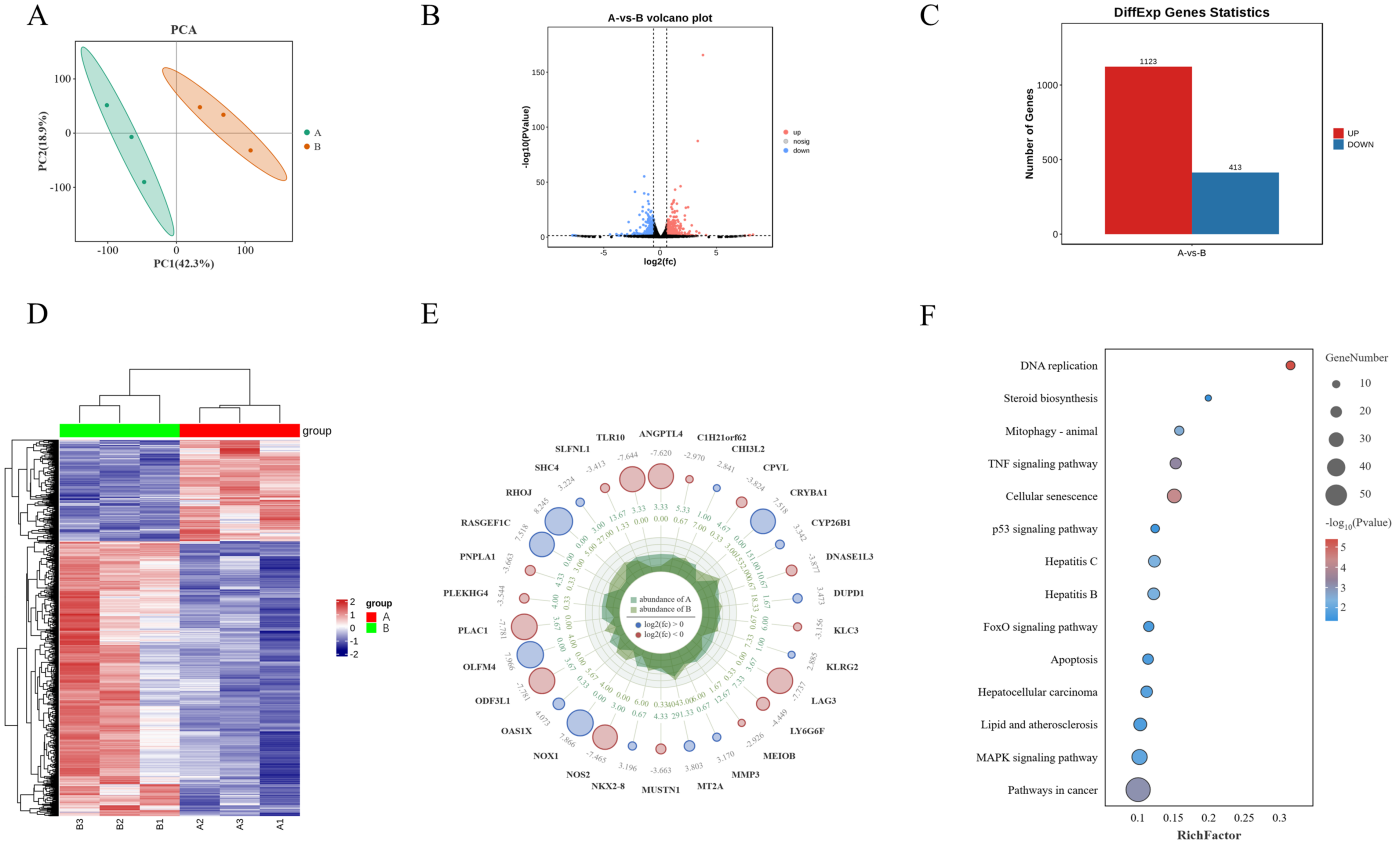
Transcriptomic analysis reveals the impact of Guiqi Yimu Powder treatment on gene expression in bovine hepatocytes. **(A)** PCA plot illustrating the overall expression pattern differences between control and Guiqi Yimu Powder-treated bovine hepatocytes. **(B)** Volcano plot depicting the relationship between the significance of differentially expressed genes and fold changes in expression. Red dots represent significantly upregulated genes, blue dots represent significantly downregulated genes, and black dots represent non-significant genes. **(C)** Bar chart showing the number of significantly upregulated and downregulated genes in the comparison groups. **(D)** Heatmap of differentially expressed genes displaying significant gene expression differences between the comparison groups. The color gradient ranges from blue (low expression) to red (high expression) indicating changes in expression levels. **(E)** Radar chart illustrating the functional distribution of the top 20 most significantly differentially expressed genes. **(F)** KEGG enrichment analysis chart showing the enrichment of differentially expressed genes with bubble size indicating the number of genes and color shading indicating the level of enrichment significance.

PCA based on expression levels showed that biological replicates within the same experimental groups clustered tightly together (PC1: 42.3% and PC2: 18.9%), and samples from different experimental groups were clearly separated along PC1, confirming the reliability and stability of the sequencing results (Figure 6A). Differential gene expression analysis identified a total of 1,536 significantly differentially expressed genes (Supplementary Table S2, *p* < 0.05), with 1,123 genes upregulated and 413 genes downregulated (Figures 6B,C). Cluster analysis was used to generate a heatmap of differentially expressed genes, which demonstrated good reproducibility within groups and clear differences between groups, indicating that Guiqi Yimu Powder treatment significantly altered the gene expression profiles in bovine hepatocytes (Figure 6D). To more clearly illustrate the significant differences between the two comparison groups, a radar chart was created, detailing the expression patterns of the top 20 differentially expressed genes (Figure 6E).

KEGG pathway enrichment analysis of the differentially expressed genes revealed several key pathways related to metabolism, inflammation and oxidative stress, and signal transduction. The analysis showed that metabolism-related pathways were mainly concentrated in steroid biosynthesis, while inflammation and oxidative stress-related pathways were enriched in lipid and atherosclerosis, hepatitis B, and hepatitis C. Signal transduction pathways included key pathways such as the p53 signaling pathway and the MAPK signaling pathway (*p* < 0.05, Figure 6F).

### 3.6 Identification and comparison of differential abundance proteins

**Figure 7:**
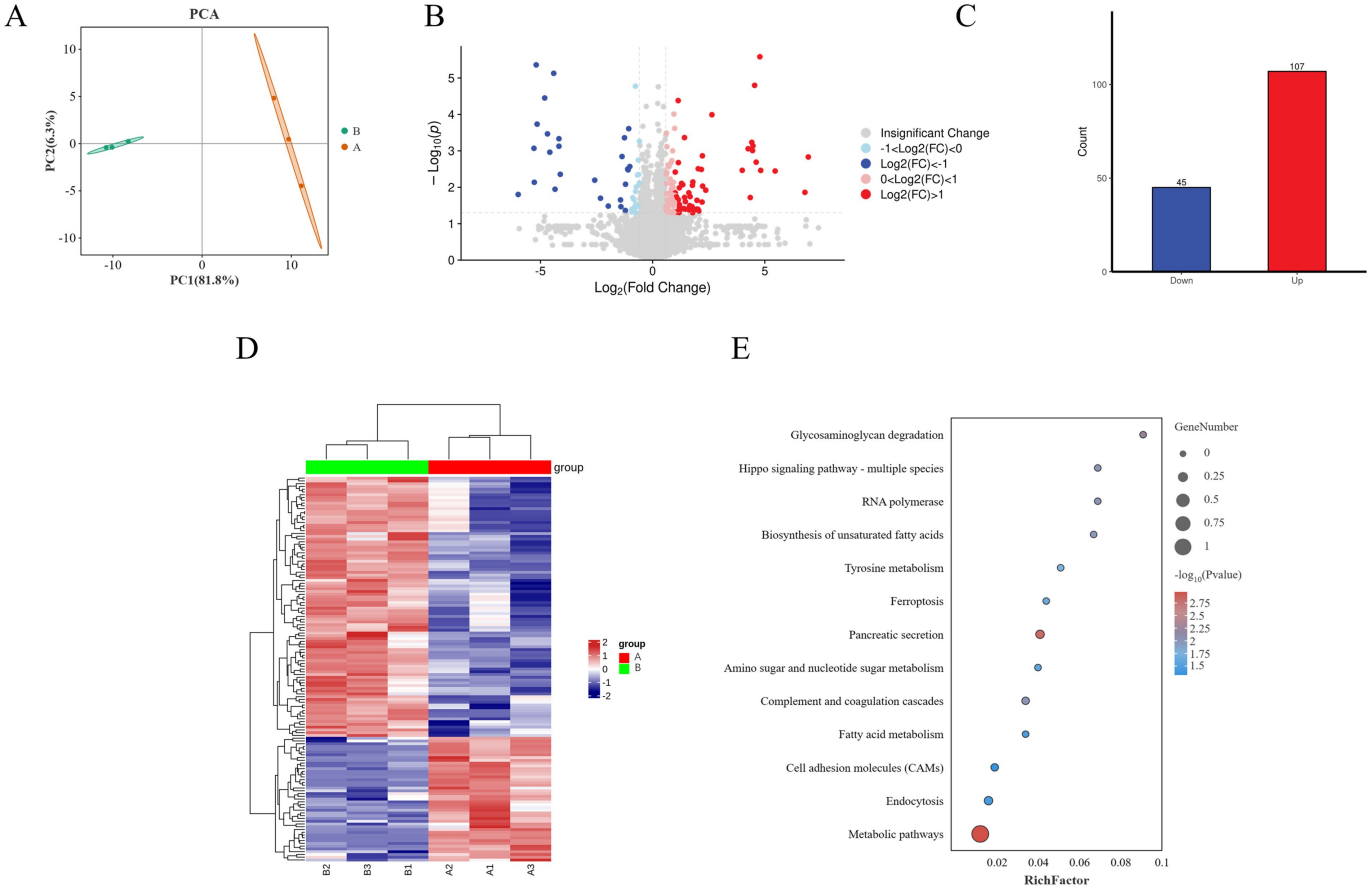
Overview of proteomics analysis showing the effects of Guiqi Yimu Powder treatment on protein expression in bovine hepatocytes. **(A)** PCA plot illustrating the overall differences in protein expression patterns between control and TMAO-treated bovine hepatocyte samples. **(B)** Volcano plot revealing the relationship between the significance of differentially expressed proteins and fold changes in expression. Red dots represent significantly upregulated proteins, blue dots represent significantly downregulated proteins, and gray dots represent non-significant proteins. **(C)** Bar chart showing the number of significantly upregulated and downregulated proteins in the TMAO-treated group. **(D)** Heatmap displaying the most significantly differentially expressed proteins between the comparison groups, with a color gradient from blue to red indicating changes in protein expression levels. **(E)** KEGG enrichment analysis revealing the enrichment of differentially expressed proteins in metabolic pathways. The size of the circles represents the number of proteins, while the color shading indicates the level of enrichment significance.

To elucidate the mechanisms by which Guiqi Yimu Powder affects lipid metabolism in bovine hepatocytes, we employed Label-Free Quantification (LFQ) proteomics to identify proteins within the cells. PCA scatter plots showed a clear separation between the TMAO-treated group and the control group along the first principal component (PC1), indicating that TMAO treatment significantly altered the overall protein expression profiles in bovine hepatocytes (Figure 7A). Subsequent differential expression analysis identified a total of 152 significantly differentially expressed proteins (Supplementary Table S3, *p* < 0.05), with 107 proteins upregulated and 45 downregulated (Figures 7B,C). A hierarchical clustering heatmap visually demonstrated the expression differences of these proteins between the different treatment groups (Figure 7D).

KEGG enrichment analysis of the differentially expressed proteins revealed that metabolism-related pathways were primarily enriched in metabolic pathways, fatty acid metabolism, and tyrosine metabolism. In contrast, inflammation and oxidative stress-related pathways were mainly enriched in ferroptosis and the complement and coagulation cascades. Pathways related to signal transduction included key pathways such as cell adhesion molecules (CAMs) and the Hippo signaling pathway (*p* < 0.05, Figure 7E).

### 3.7 Identification and comparison of differential metabolites

**Figure 8:**
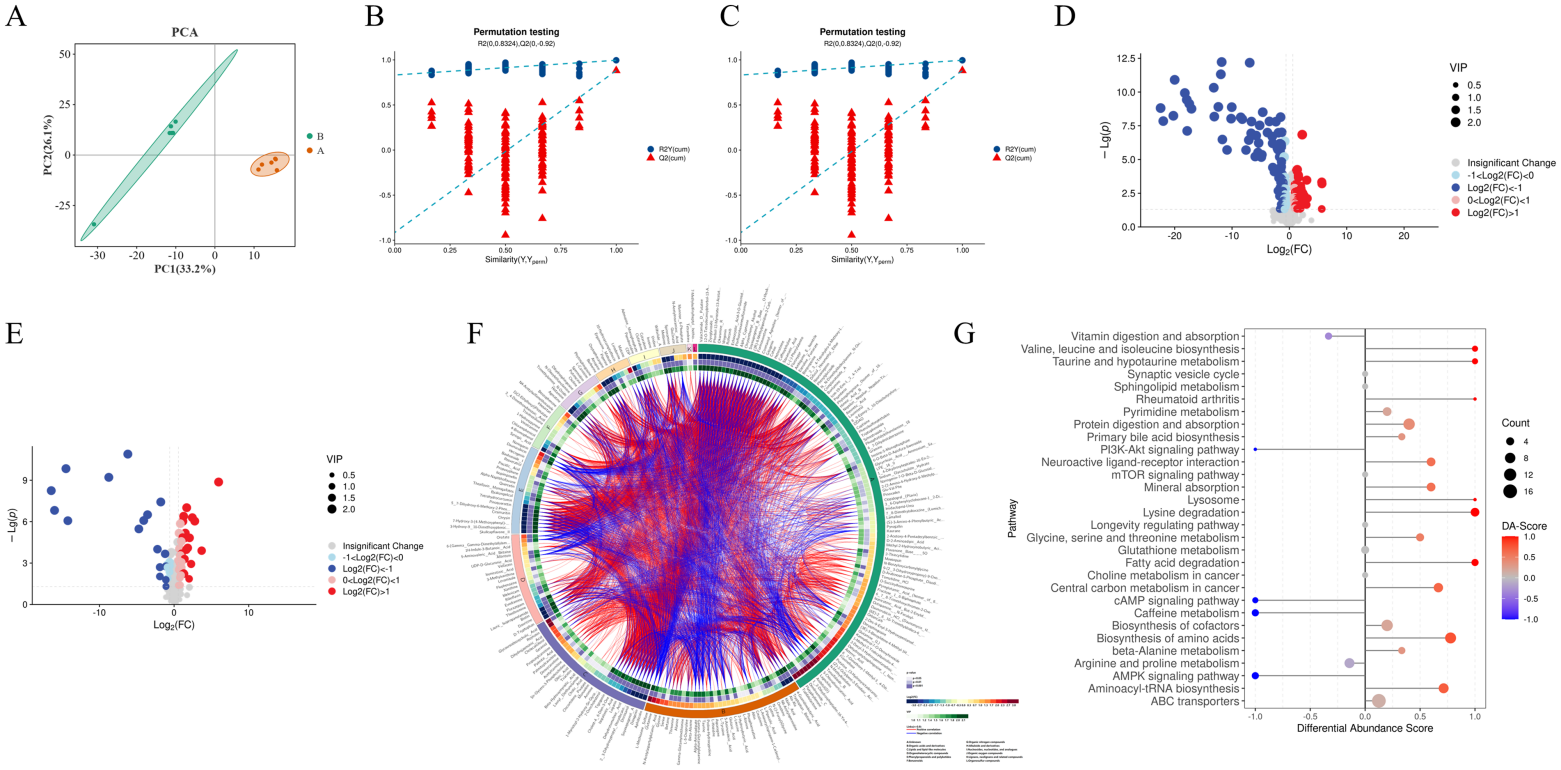
Analysis of the impact of Guiqi Yimu Powder treatment on metabolic profiles in bovine hepatocytes **(A)** PCA plot illustrating the overall distribution and variability in metabolic levels between the comparison groups. **(B,C)** Quality parameter plots of Orthogonal Partial Least Squares Discriminant Analysis (OPLS-DA) models in positive and negative ion modes, providing information on model fitting quality and predictive capability, including R^2^ and Q^2^ values. **(D,E)** Volcano plots in positive and negative ion modes depicting the relationship between the significance of metabolites and fold changes, with red and blue dots indicating significantly upregulated and downregulated metabolites, respectively. **(F)** Heatmap of differential metabolites showing the differences in metabolite expression levels between bovine hepatocyte samples from the comparison groups. The color gradient from blue (low expression) to red (high expression) indicates changes in metabolite abundance, representing the relative expression levels of different metabolites across samples. **(G)** DA score results presenting the overall trend of metabolites in pathways compared to the control group. The x-axis represents the overall trend changes of all metabolites in the pathway, and the y-axis denotes the pathways. The size of the circles indicates the number of metabolites annotated to that pathway, while the color gradient from blue to red indicates DA scores ranging from −1 to 1. A DA score of −1 indicates that the abundance of all metabolites in the pathway has decreased, while a score of 1 indicates that the abundance of all metabolites has increased. The closer the DA score is to 1 or −1, the more the overall expression in that pathway tends toward upregulation or downregulation, respectively.

To further investigate the metabolic mechanisms by which Guiqi Yimu Powder affects bovine hepatocytes, we performed untargeted metabolomics analysis on samples from the experimental and control groups. PCA revealed significant differences in metabolic profiles between the two groups, with good biological reproducibility within the groups (Figure 8A). The OPLS-DA score plots indicated clear separation between the groups in both positive and negative ion modes. The OPLS-DA model was validated using 200 permutation tests, yielding model quality parameters of R^2^Y = 0.996 and Q^2^ = 0.882 for positive ion mode, and R^2^Y = 0.999 and Q^2^ = 0.894 for negative ion mode, confirming the model’s high stability and lack of overfitting (Figures 8B,C). Differential analysis identified 259 significantly different metabolites, with 109 upregulated and 150 downregulated (Figures 8D,E). These differential metabolites were further subjected to cluster analysis (Figure 8F).

KEGG metabolic pathway enrichment analysis of the 259 differential metabolites was conducted to explore the biological pathways potentially affected by Guiqi Yimu Powder in bovine hepatocytes (Supplementary Table S4). The results indicated that metabolism-related pathways were primarily enriched in glutathione metabolism, fatty acid degradation, choline metabolism, amino acid biosynthesis, as well as the metabolism of glycine, serine, and threonine. Pathways related to inflammation and oxidative stress were mainly enriched in rheumatoid arthritis and various cancer pathways. Signal transduction pathways included key pathways such as the mTOR signaling pathway and the PI3K-Akt signaling pathway (*p* < 0.05, Figure 8G).

### 3.8 Integrated multi-omics data analysis

**Figure 9:**
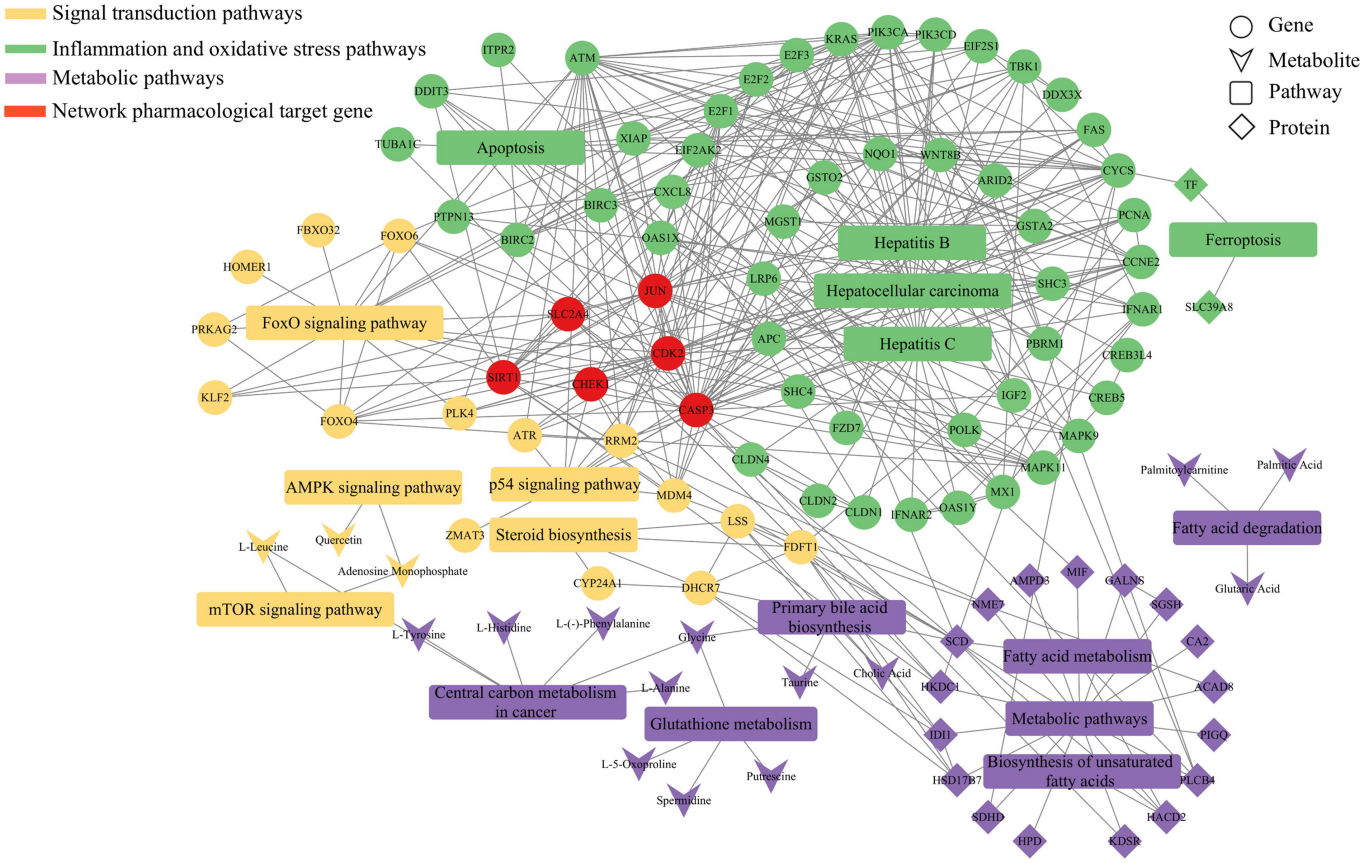
Metabolic regulatory network of Guiqi Yimu Powder on bovine hepatocytes revealed by multi-omics data analysis. This figure illustrates the lipid metabolism regulatory network constructed by integrating proteomics, transcriptomics, and metabolomics data. The nodes in the network represent different categories, including genes, proteins, metabolites, and pathways, while the edges indicate the interactions or regulatory relationships between these molecules.

**Figure 10:**
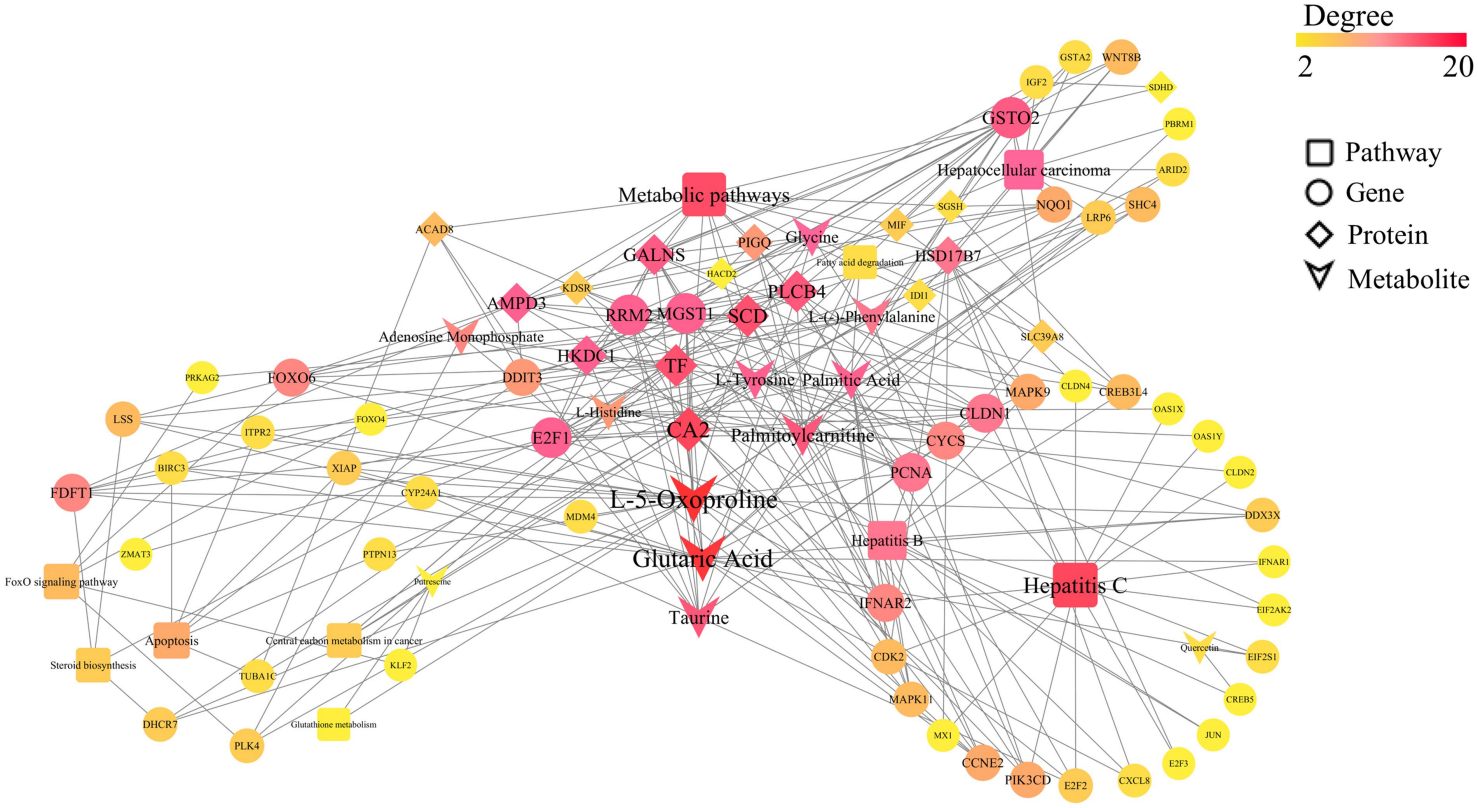
Multi-omics Pearson correlation network diagram. This figure presents the interaction network among key genes, proteins, and metabolites in bovine hepatocytes treated with Guiqi Yimu Powder, based on Pearson correlation analysis results. The node types within the network represent different categories, including genes, proteins, metabolites, and pathways. The edges illustrate various regulatory relationships, with dashed lines indicating highly significant correlations (*p* < 0.01) and solid lines representing direct regulatory interactions. The color and size of the nodes reflect their relative importance within the network.

By integrating transcriptomics, proteomics, and metabolomics data, and analyzing the significantly enriched pathways (*p* < 0.05), we constructed a comprehensive regulatory network. As shown in Figure 9, this network revealed the multi-faceted impact of Guiqi Yimu Powder treatment on TMAO-induced metabolic pathways in bovine fatty liver cells. The results indicated that Guiqi Yimu Powder significantly affected signal transduction pathways, lipid metabolism pathways, and pathways related to inflammation and oxidative stress in bovine hepatocytes. Target genes identified through network pharmacology analysis were mainly concentrated in inflammation pathways, underscoring the critical roles these genes play in the pathological mechanisms. To further explore the interactions among key genes, proteins, and metabolites that showed significant changes upon Pearson correlation analysis was performed on Guiqi Yimu Powder treatment in bovine hepatocytes, and a correlation network diagram was subsequently constructed (Figure 10). The analysis revealed that L-5-Oxoproline showed a highly significant correlation with many key genes and proteins (*p* < 0.01), which were significantly enriched in signal transduction pathways, lipid metabolism pathways, and inflammation-related pathways (*p* < 0.05). The significant increase in L-5-Oxoproline represents a crucial metabolic alteration, highlighting its potential regulatory role in signal transduction, lipid metabolism, and the modulation of inflammation and oxidative stress.

**Figure 11:**
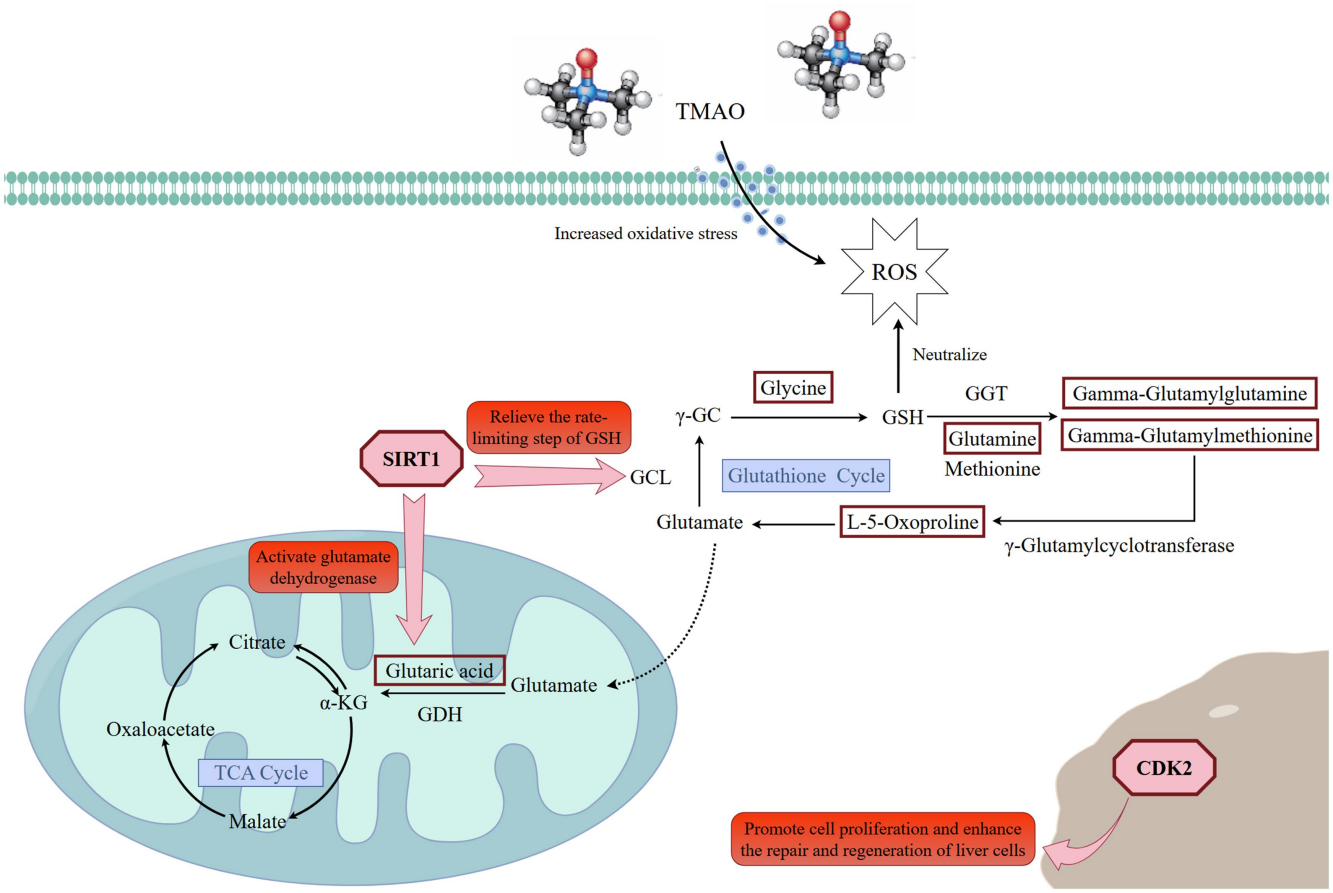
Regulatory mechanisms of Guiqi Yimu Powder on glutathione cycle, oxidative stress, and energy metabolism. The red rectangles in the figure represent significantly upregulated differential metabolites. Created with Figdraw (https://www.figdraw.com).

On the other hand, the key genes and proteins significantly enriched in these pathways suggested that L-5-Oxoproline acted as a central node mediating the interaction and coordination of multiple metabolic pathways. For instance, significant associations were observed between L-5-Oxoproline and several related genes and proteins: lanosterol synthase (*LSS*) and squalene synthase (*FDFT1*), which are involved in lipid metabolism regulation; microsomal glutathione S-transferase 1 (*MGST1*) and X-linked inhibitor of apoptosis protein (*XIAP*), which are involved in oxidative stress regulation; interferon alpha/beta receptor 2 (*IFNAR2*), which is involved in inflammatory responses; and low-density lipoprotein receptor-related protein 6 (*LRP6*), which is important in energy metabolism. Investigating these targets further can provide deeper insights into the potential mechanisms of Guiqi Yimu Powder in treating bovine fatty liver. Finally, through comprehensive multi-omics data analysis, we elucidated the potential mechanisms by which Guiqi Yimu Powder affects bovine fatty liver cells. Figure 11 illustrated how Guiqi Yimu Powder regulated bovine fatty liver through multiple pathways. Specifically, in TMAO-induced fatty liver formation, oxidative stress and damage lead to glutathione depletion. Guiqi Yimu Powder regulated the development of bovine fatty liver by affecting glutathione metabolism and energy metabolism. By significantly upregulating metabolites such as L-5-Oxoproline, Glutamine, Gamma-Glutamylmethionine, Gamma-Glutamylglutamine, and Glycine, Guiqi Yimu Powder enhances glutathione synthesis, thereby improving cellular antioxidant capacity. Concurrently, L-5-Oxoproline converted to glutamate, which further converts to alpha-ketoglutarate (*α*-KG, an important intermediate in the TCA cycle) and succinate, promoting the efficiency of the TCA cycle and boosting energy metabolism levels. Additionally, the upregulation of *SIRT1* played a crucial role in this mechanism. *SIRT1* can upregulate the expression of multiple genes involved in glutathione metabolism, including glutamate-cysteine ligase (GCL), which is the rate-limiting enzyme in glutathione synthesis. *SIRT1* also promotes the conversion of glutamate to α-KG, further enhancing the TCA cycle and energy metabolism. Furthermore, the upregulation of the target gene *CDK2* was beneficial in this context. *CDK2* was involved in cell cycle regulation and can aid in cellular repair and regeneration, contributing to the maintenance and restoration of tissue structural integrity. This integrated mechanism ultimately contributed to the establishment and maintenance of tissue structural integrity, improving the overall health of hepatic cells.

## 4 Discussion

Fatty liver is a pathological condition resulting from the excessive accumulation of fat within the liver, involving complex metabolic regulatory mechanisms, such as fatty acid synthesis, storage, *β*-oxidation, and conversion into TG. Additionally, inflammatory responses and oxidative stress play crucial roles in the onset and progression of fatty liver. In recent years, the application of Guiqi Yimu Powder and its compound formula in livestock has garnered increasing attention. The related literature reports that this compound demonstrates significant efficacy in regulating metabolic balance, enhancing immunity, and exhibiting anti-inflammatory and antioxidant properties. However, the mechanisms by which Guiqi Yimu Powder regulates lipid metabolism in dairy cows remain unclear. In this study, we systematically explored the therapeutic effects of Guiqi Yimu Powder in a TMAO-induced fatty liver model. We found that Guiqi Yimu Powder significantly alleviated hepatic cell damage and lipid droplet accumulation caused by TMAO. Subsequent integrative multi-omics analysis revealed that L-5-Oxoproline, a key intermediate in maintaining the GSH cycle, occupied a central position in the metabolic network. Guiqi Yimu Powder treatment significantly upregulated L-5-Oxoproline (FC = 2.6), thereby enhancing the GSH cycle. Additionally, L-5-Oxoproline was converted to glutamate, which was then further converted to *α*-KG, an important intermediate in the TCA cycle, and succinate (FC = 1.8), This process enhanced the efficiency of the TCA cycle and increased energy metabolism levels. Further analysis showed that *SIRT1* (FC = 1.5) was upregulated, leading to the upregulation of various genes involved in glutathione metabolism, such as GCL, the rate-limiting enzyme in glutathione synthesis. Additionally, *SIRT1* facilitated the conversion of glutamate to α-ketoglutarate (α-KG). Additionally, Guiqi Yimu Powder upregulated the expression of target gene *CDK2* (FC = 1.6), potentially playing a role in cellular proliferation and metabolic regulation mechanisms. These findings suggested that Guiqi Yimu Powder had potential therapeutic prospects in the multi-target and multi-pathway regulation of fatty liver pathology.

In high-yield dairy herds, the high energy demands postpartum trigger the release of substantial amounts of non-esterified fatty acids (NEFA) from adipose tissue into the bloodstream. However, the liver’s capacity for fatty acid oxidation is limited and cannot fully process these excessive fatty acids, leading to lipid accumulation within hepatocytes (40–43). When the liver uptakes excessive fatty acids, metabolic pressure increases, resulting in elevated levels of reactive oxygen species (ROS) and consequently inducing oxidative stress. Oxidative stress damages lipid membranes, proteins, and DNA within hepatocytes, further exacerbating lipid accumulation and inflammation in the liver. Glutathione is considered one of the most important intracellular antioxidants. By directly scavenging reactive oxygen species (ROS), glutathione protects cells from oxidative damage and plays a crucial role in maintaining liver health (44). Increasing glutathione levels can effectively mitigate oxidative stress associated with fatty liver and enhance the antioxidant capacity of liver cells (45). According to Parvin et al., GSH depletion and reduced GSH-dependent antioxidant activity, along with leukocyte accumulation and hepatic inflammation, are major sources of excessive ROS production in non-alcoholic fatty liver disease (NAFLD) (46). Mariapia et al. also highlighted that GSH is not only a free radical scavenger but also regulates networks of cell survival, necrosis, and apoptosis, impacting signal transduction and transcription factor functions. They emphasized the close correlation between GSH levels and the progression of liver inflammatory diseases (47). In the glutathione synthesis pathway, GCL catalyzes the formation of *γ*-glutamylcysteine from glutamate and cysteine. This is the first step and the rate-limiting step of glutathione synthesis (48, 49). Numerous studies have demonstrated that the target gene SIRT1 upregulates the expression of the GCL gene by deacetylating FOXO3a, which can significantly enhance the coupling rate of glutamate and cysteine, thereby increasing the overall yield of glutathione (50, 51). Glutathione is primarily synthesized and cycled within cells through specific pathways. Glutamine is converted to glutamate by glutamine synthetase (GS), and then glutamate and L-5-Oxoproline are generated under the action of glutamine transaminase (GGT). L-5-Oxoproline is subsequently converted to glutamate by 5-oxoprolinase (OPase). Glutamate is then coupled with cysteine and glycine to finally synthesize glutathione (52–54). In this study, the intermediate metabolites involved in the GSH cycle, including L-5-Oxoproline, glycine, glutamine, gamma-glutamylglutamine, and gamma-glutamylmethionine, were significantly upregulated, and the target gene SIRT1 was notably upregulated as well. Thus, we hypothesize that Guiqi Yimu Powder treatment in bovine fatty liver cells helps maintain the glutathione cycle, promote glutathione synthesis, and thereby enhance cellular antioxidant capacity. Energy metabolism imbalance is another critical factor leading to bovine fatty liver disease, which typically occurs during the periparturient and early lactation periods. During these times, cows require substantial energy to meet lactation demands and maintain physical strength. Due to the high energy demands, dairy cows often experience a negative energy balance. This negative energy balance can induce insulin resistance, disrupting glucose metabolism and exacerbating energy metabolism imbalance. The insufficient glucose and elevated NEFA drive the liver to preferentially utilize fatty acid oxidation to generate energy. However, this process consumes a considerable amount of NAD+ and can potentially disrupt or overload the TCA cycle. Heather’s study suggests that when the amount of acetyl-CoA available for oxidation exceeds the TCA cycle’s capacity, it contributes to fatty liver in cows experiencing periparturient negative energy balance. They further emphasized that enhancing the TCA cycle might help balance the oxidation and esterification of acetyl-CoA, potentially alleviating the development of fatty liver (55). In our study, the significant upregulation of L-5-Oxoproline increased the supply of glutamate, which is further converted to *α*-ketoglutarate and succinate. α-Ketoglutarate is a vital intermediate of the TCA cycle, providing energy for cells and participating in various anabolic and catabolic processes, thereby improving cellular energy metabolism efficiency. By enhancing the activity of the TCA cycle, cells can more efficiently produce energy and increase ATP yield. Additionally, SIRT1 activates several key enzymes related to the TCA cycle and amino acid metabolism through deacetylation, including glutamate dehydrogenase (GLUD1), thereby improving the efficiency of converting glutamate to *α*-KG (56, 57). This is directly beneficial in situations of energy deficiency or increased metabolic stress, aligning with Heather’s findings. Similarly, *CDK2*, a key regulator of the cell cycle, showed significant upregulation. *CDK2* plays a pivotal role in the transition from the G1 phase to the S phase, facilitating DNA replication and cell division (58). Its upregulation promotes cell proliferation, which is critical for liver regeneration and repair. Furthermore, upregulated *CDK2* expression has been linked to improved cellular responses to DNA damage, supporting hepatocyte survival and function. Studies suggest that elevated *CDK2* levels are often associated with enhanced cellular repair mechanisms and better outcomes in liver health (14). The significant upregulation of *CDK2* in liver tissues may therefore contribute to maintaining hepatic cellular integrity and function, providing a protective effect against liver pathologies.

Although we predicted that Guiqi Yimu Powder treatment might enhance the GSH cycle by increasing intermediate metabolites such as L-5-Oxoproline, our results showed no significant changes in the key metabolites glutamate and GSH. This could be attributed to several mechanisms. First, metabolic flux redistribution may play a role. Even though intermediate metabolites like L-5-Oxoproline were upregulated, glutamate did not show a significant increase, possibly because glutamate was rapidly consumed in downstream metabolic pathways within the cells. For example, while glutamate can be used for GSH synthesis, it can also be converted into other important metabolites such as α-ketoglutarate (59, 60). Thus, even if upstream intermediates increase, the levels of downstream products might not rise significantly due to their rapid utilization. Additionally, GSH undergoes rapid turnover during the antioxidation process, being converted to oxidized glutathione (GSSG) and then reduced back to GSH (61). Therefore, measurements at specific time points may not capture its dynamic changes. Even if the synthesis rate of GSH increases, the steady-state level of intracellular GSH might not appear significantly elevated because it is quickly utilized to counteract oxidative stress. Second, competition from other metabolic pathways could be a factor. Glutamate has multiple metabolic roles, such as participating in energy metabolism, synthesizing other amino acids, or producing neurotransmitters (62, 63). Consequently, the upregulated precursor substances might be diverted to other metabolic pathways, leading to no significant increase in glutamate and GSH levels. Andres et al. demonstrated that overexpression of 5-oxoprolinase, which reduces 5-Oxoproline (pyroglutamate) concentration, could improve cardiac function post-myocardial infarction in mice. In their study, 5-Oxoproline concentrations were elevated in both heart and liver tissues of myocardial infarction mice, and the GSH/GSSG ratio in heart tissue significantly decreased, whereas such changes were not observed in liver tissue and body fluids. This indicates that strong oxidative stress was present in heart tissue, and despite the increase in 5-Oxoproline, GSH levels did not significantly rise due to rapid turnover and consumption to counteract oxidative stress (64). This conclusion aligns with our findings. Therefore, while Guiqi Yimu Powder treatment may have an indirect effect on the GSH cycle by increasing intermediate metabolites like L-5-Oxoproline, glutamate and GSH did not show significant upregulation under specific experimental conditions due to the aforementioned mechanisms. These results do not negate the potential effects of Guiqi Yimu Powder but suggest that further research is needed to elucidate its specific metabolic regulatory mechanisms within cells. Moreover, an increase in abundance does not necessarily correspond to enhanced function. Future research should focus on other methods and indicators to demonstrate enhanced antioxidant capacity, such as directly measuring antioxidant enzyme activity or changes in oxidative stress markers. Through these approaches, we can gain a more comprehensive understanding of the role of Guiqi Yimu Powder in cellular metabolism and oxidative stress response.

In summary, Guiqi Yimu Powder, as a traditional Chinese herbal formula, can alleviate fatty liver symptoms in dairy cows through multiple pathways, including antioxidative, anti-inflammatory, and energy metabolism regulation. The active components in Guiqi Yimu Powder can enhance the GSH cycle by increasing L-5-Oxoproline, thereby reducing reactive ROS levels and mitigating oxidative stress damage to hepatocytes. This process also inhibited the expression and release of inflammatory factors. Additionally, L-5-Oxoproline can be converted into glutamate, which subsequently participates in the tricarboxylic acid (TCA) cycle to promote energy production. Therefore, through a comprehensive range of actions, Guiqi Yimu Powder helps mitigate fatty liver symptoms in dairy cows, improving their overall health and production performance.

## 5 Conclusion

In this study, we developed a dairy cow fatty liver cell model using TMAO and integrated data from transcriptomics, proteomics, and metabolomics. This approach helped us uncover how Guiqi Yimu Powder affects bovine hepatocytes. Our analysis highlighted the key role of L-5-Oxoproline in the glutathione cycle, influencing antioxidation, energy metabolism, and anti-inflammatory processes. The active components in Guiqi Yimu Powder help improve fatty liver conditions by boosting glutathione levels, balancing energy metabolism, and reducing inflammation. This research enhances our understanding of metabolic diseases in dairy cows, offering insights into the prevention and treatment of bovine fatty liver.

## Data Availability

The datasets presented in this study can be found in online repositories. The names of the repository/repositories and accession number(s) can be found in the article/Supplementary material.

## Footnotes

^1^ https://old.tcmsp-e.com/tcmsp.php

^2^ https://cn.string-db.org

^3^ https://www.omicshare.com

^4^ http://www.kegg.jp/

^5^ https://www.omicshare.com
